# Supplementary material for: Comprehensive Annotation and Functional Exploration of MicroRNAs in Lettuce
Source: Front Plant Sci. 2021 Dec 24;12:781836. doi: 10.3389/fpls.2021.781836 (PMC8739914; doi:10.3389/fpls.2021.781836)
Supplement: Supplementary file 2 [file Data_Sheet_8.ZIP › GSM2230747.plot/Lsa-miRN1690_Lsat_1_v5_gn_2_86861.1_65_TPlot.pdf]

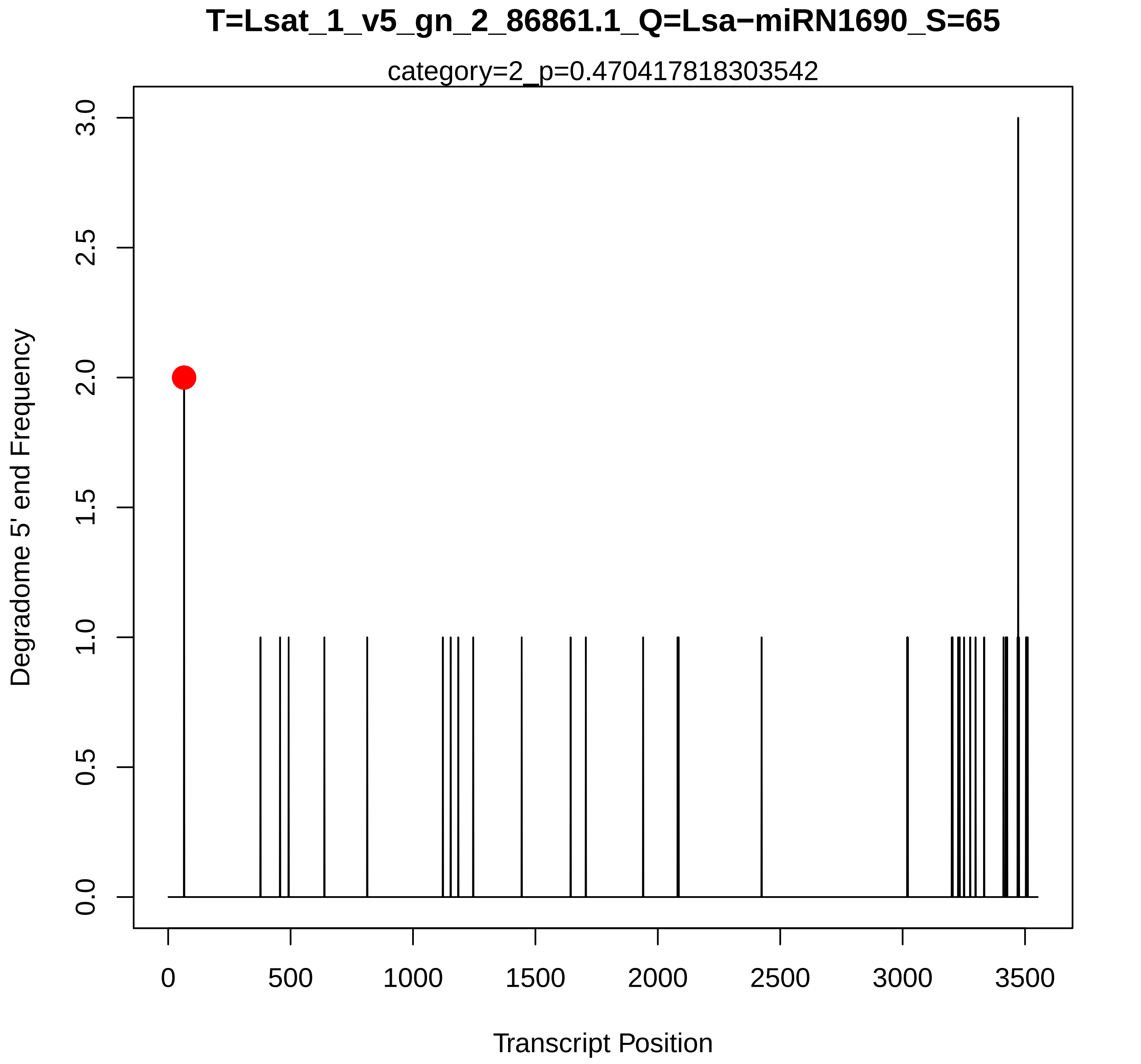

T=Lsat_1_v5_gn_2_86861.1_Q=Lsa−miRN1690_S=65
category=2_p=0.470417818303542
Degradome 5' end Frequency
0.0
0.5
1.0
1.5
2.0
2.5
3.0
0
500
1000
1500
2000
2500
3000
3500
Transcript Position
